# Identification and expression of the *Di19* gene family in response to abiotic stress in common bean (*Phaseolus vulgaris* L.)

**DOI:** 10.3389/fgene.2024.1401011

**Published:** 2024-05-30

**Authors:** Wei Guo, Xinhui Li, Tao Yang, Chunguo Huang, Bo Zhao, Peng Wang

**Affiliations:** ^1^ Department of Basic Sciences, Shanxi Agricultural University, Taigu, China; ^2^ Shanxi Houji Laboratory, College of Agriculture, Shanxi Agricultural University, Taigu, China

**Keywords:** common bean, Di19, gene family, gene expression, abiotic stress

## Abstract

Drought-induced 19 (Di19) protein plays critical biological functions in response to adversity as well as in plant growth and development. Exploring the role and mechanism of *Di19* in abiotic stress responses is of great significance for improving plant tolerance. In this study, six *Di19* genes were identified in the common bean (*Phaseolus vulgaris* L.), which were mainly derived from segmental duplications. These genes share conserved exon/intron structures and were classified into three subfamilies based on their phylogenetic relationships. The composition and arrangement of conserved motifs were consistent with their phylogenetic relationships. Many hormone- and stress-responsive elements were distributed in the promoters region of *PvDi19* genes. Variations in histidine residues in the Cys2/His2 (C2H2) zinc-finger domains resulted in an atypical tertiary structure of PvDi19-5. Gene expression analysis showed rapid induction of *PvDi19-1* in roots by 10% PEG treatment, and *PvDi19-2* in leaves by 20% PEG treatment, respectively. Most *PvDi19s* exhibited insensitivity to saline-alkali stress, except for *PvDi19-6*, which was notably induced during later stages of treatment. The most common bean *Di19* genes were inhibited or not regulated by cadmium stress, but the expression of *PvDi19-6* in roots was significantly upregulated when subjected to lower concentrations of cadmium (5 mmol). Moreover, *Di19s* exhibited greater sensitivity to severe cold stress (6°C). These findings enhance our understanding of the role of *PvDi19s* in common bean abiotic stress responses and provide a basis for future genetic enhancements in common bean stress tolerance.

## 1 Introduction

Environmental pressures caused by climate change and population growth have adverse implications for agriculture and pose a growing problem for food security (Gong et al., 2020). Various abiotic stresses, including drought, soil salinity, heavy metal toxicity, and extreme climates (e.g., low and high temperatures), cause a wide range of changes in plant morphology, physiology, biochemistry, and cellular processes (Zhang et al., 2020; Zhang et al., 2022), and are eventually responsible for reduced agricultural productivity (Chang et al., 2020). Plants develop adaptive responses to abiotic stresses through complex and elaborate signaling networks, which are controlled by multiple genes, especially dynamic changes in the expression levels of stress-responsive genes (Liu et al., 2022).

It is well known that transcription factors (TFs), as DNA-binding proteins, can bind specifically to cis-acting elements in gene promoter regions in eukaryotes. They can perceive stress signals and regulate the expression of numerous downstream genes and have been confirmed to play critical roles in plant growth and development, morphogenesis, and resistance to abiotic stresses (Singh et al., 2002; Chen and Zhu, 2004; Sakamoto et al., 2004). Zinc finger proteins (ZFPs) are a major TF family in plants (Takatsuji, 1999; Englbrecht et al., 2004), in which Zn ions act as binding centers that are tetrahedrally coordinated with repeating histidine (His) and/or cysteine (Cys) residues in multiple permutations by hydrophobic (Li G. et al., 2010; Salih et al., 2019; Zhong et al., 2020). Drought-induced 19 (Di19) proteins belong to the Cys2/His2 (C2H2) class of ZFPs, which are encoded by a small gene family containing two unusual C2H2-type zinc finger structural domains that are evolutionarily well-conserved (Milla et al., 2006). Numerous studies have shown that Di19 proteins play important biological roles in plant growth and development, as well as in response to adversity (Li G. et al., 2010; Li S. et al., 2010; Liu et al., 2013).

Di19 proteins were first identified in *Arabidopsis*, and are involved in an ABA-independent signaling pathway in response to drought stress (Gosti et al., 1995; Zhao J. et al., 2017). *AtDi19-1* and *AtDi19-3* are rapidly induced by drought stress, and the overexpression of *AtDi19-3* increases susceptibility to drought (Qin et al., 2014). The expression of *AtDi19-2* and *AtDi19-4* is strongly upregulated only by high-salinity stress. *AtDi19-5* may be transiently upregulated in roots under salt stress (Milla et al., 2006). Notably, *AtDi19-7* does not respond to abiotic stress but is involved in regulation of the optical signal pathway (Kang et al., 2005). In addition, the transactivation activity of AtDi19-1 can be enhanced through phosphorylation by interacting with CPK11 (a calcium-binding protein kinase) *in vitro* (Milla et al., 2006). Furthermore, Liu et al. (2013) reported that *AtDi19* directly upregulates the expression of pathogenesis-related genes (*PR1*, *PR2*, and *PR5*) by binding to the TACA (A/G)T element in promoter regions and responding to drought stress.

To date, members of the *Di19* family of genes have been identified in various plant species, the functions of which have been extensively studied, and almost all of them are closely related to abiotic stress (Li G. et al., 2010; Liu et al., 2013; Qin et al., 2014). For example, the expression of *TaDi19A* is significantly upregulated by salinity, osmotic, and cold stresses in wheat, the overexpression of *TaDi19A* in *Arabidopsis* causes plants to exhibit a more salt-sensitive phenotype, ABA, and osmotic stresses (Li S. et al., 2010). In soybean, *GmDi19-5* is involved in responses to abiotic stresses, and is induced by salt, drought, ABA, oxidative stress, and high-temperature stress. GmDi19-5 protein is involved in interactions with GmLEA3.1 and GmDnaJ (Feng et al., 2015; Zhao Y. et al., 2017). In cotton, both *GhDi19-1* and *GhDi19-2* may be involved in responses to salt and drought stress and ABA signaling during the early stages of plant development (Li G. et al., 2010; Wang et al., 2014). In maize, *Z*There are five *Di19-like* genes in rice, among which *OsDi19-4* is phosphorylated by OsCDPK14, and which positively regulates ABA stress by regulating the expression of ABA-related genes*mDi19-1* expression is induced by PEG and NaCl stress. *Arabidopsis* Z*mDi19-1-*overexpressing lines exhibit enhanced tolerance to salt stress (Zhang et al., 2019). Furthermore, phenotypic and metabolomic analyses have revealed the role of *Di19* in determining acquired drought tolerance in rice (Sankarapillai et al., 2023). A recent study concerning the function of wheat *Di19* in plant growth and development indicated that *TaDi19-7* overexpression in *Arabidopsis* significantly promotes flowering and branching in transgenic plants (Du et al., 2023). These findings indicate that different members of the *Di19* family may respond to different signaling stimuli and accomplish specific functions.

Common bean (*Phaseolus vulgaris* L.) is an important food legume worldwide. In developing countries in Asia and Africa, it is an important source of nutrition and protein for people (Joshi et al., 2017; Li et al., 2023). However, their growth and yields are severely inhibited by various abiotic stresses (Chen et al., 2008; Beaver and Osorno, 2009; Zhang et al., 2023). To date, detailed information regarding the *Di19* gene family in common bean, especially the expression patterns and roles of *PvDi19* genes in various abiotic stress responses, is rarely reported. Here, the *Di19* gene family in common bean was investigated by identifying its seven members and their chromosomal locations, as well as by analyzing their phylogenetic relationships, gene structures, conserved domains, and tertiary structure. Furthermore, expression analysis of *PvDi19* genes was performed under various abiotic stresses, including PEG, saline-alkali, cadmium (Cd), and cold. This study provides a basis for elucidating the mechanism of stress regulation by *Di19* TFs in common bean.

## 2 Materials and methods

### 2.1 Identification and characterization of *Di19* family members in common bean

The reference genome sequences for common bean and other seven species including *Arabidopsis* (*Arabidopsis thaliana*), barrel medic (*Medicago truncatula*), soybean (*Glycine max*), chickpea (*Cicer arietinum*), tepary bean (*Phaseolus acutifolis*), maize (*Zea mays*), and rice (*Oryza sativa*) were obtained from the Phytozome v.13 database (https://phytozome-next.jgi.doe.gov/), and the reference genome sequence of mung bean (*Vigna radiata*) was obtained from EnsemblPlants (https://plants.ensembl.org/). To identify Di19 proteins in these species, *Arabidopsis* Di19 proteins (Di19-1 to Di19-7) were used as reference queries in BLASTP searches with an e-value of 1e^−5^ and identity of 50% as the thresholds (Milla et al., 2006). Conserved domains of candidate Di19 proteins were checked using the Hidden Markov Model (HMM) profiles of zf-Di19 (PF05605) and Di19_C (PF14571) from the Pfam database (http://pfam.xfam.org/). Finally, the *Di19* gene family was named based on its chromosomal location after eliminating redundant genes and different transcripts.

The common bean *Di19* gene structures and the biochemical parameters of these proteins were obtained by GSDS (http://gsds.gao-lab.org/) with the genome annotation gff3 file and computer pI/Mw tool (https://web.expasy.org/compute_pi/), respectively (Gasteiger et al., 2005). The subcellular localization of these proteins was predicted using LocTree 3 (https://www.rostlab.org/services/loctree3/) with default parameters (Goldberg et al., 2014).

### 2.2 Chromosomal localization and collinearity analysis

The distribution of common bean *Di19* genes on chromosomes was displayed using TBtools based on gene annotation files (.gff3) (Chen et al., 2020). To investigate duplication events involving the *Di19* gene family in the common bean, collinearity analysis was performed using MCscanX on the genomes of common bean and other species. The ratio of non-synonymous mutations (Ka) to synonymous substitutions (Ks) (Ka/Ks) between these duplicated genes was calculated using KaKs_Calculator 2.0 (Wang et al., 2010).

### 2.3 Phylogenetic analysis and multiple sequence alignment

A phylogenetic tree was constructed using Di19 protein sequences from nine species, including the common bean, *Arabidopsis*, barrel medic, soybean, mung bean, chickpea, tepary bean, maize, and rice. A maximum likelihood (ML) tree was constructed using Clustal Omega and visualized using EVOLVIEW (https://www.evolgenius.info/evolview-v2/). Multiple sequence alignment of the common bean Di19 protein sequence was conducted using ClustalW and the results were visualized using Jalview software and Weblogo online software (https://weblogo.berkeley.edu/logo.cgi).

### 2.4 Gene structure and motif compositional analysis

The exon/intron organization of *Di19* gene family members from the nine species were identified using TBtools software based on CDS and genome sequences. Conserved protein motifs were identified using MEME online software (http://meme-suite.org/) (Bailey et al., 2009), with a maximum number of ten motifs and the rest of the modes followed a classic model. Motif composition was visualized using TBtools.

### 2.5 Prediction of tertiary structure and phosphorylation site

The tertiary structures of all common bean Di19 proteins were predicted using SwissModel (https://swissmodel.expasy.org/interactive) and visualized using PyMOL software. The phosphorylation sites of all PvDi19 proteins were predicted using the NetPhos 3.1 server (https://services.healthtech.dtu.dk/services/NetPhos-3.1/).

### 2.6 Cis-acting element and spatiotemporal expression pattern analysis

To analyze cis-acting elements of gene promoter regions, sequences 2, 000 bp upstream were extracted from the genome sequence and predicted using TBtools software. Afterwards, the different types of cis-acting elements were modified using Adobe illustrator. Additionally, the expression data (FPKM value matrix) of common bean *Di19* genes in different tissues and at different developmental stages were obtained from the Phytozome v.13 database (https://phytozome-next.jgi.doe.gov/).

### 2.7 Common bean stress treatments

Common bean cultivar “Pin jin yun No. 4”, with improved yield performance, which widely cultivated in Shanxi province of China and bred by Shanxi Agricultural University, was used in this study. Common bean seeds were germinated in vermiculite at 25°C for 3 days. Stress treatments were applied as followed after the first ternary compound leaf was unfolded (Li et al., 2020; Staniak et al., 2021; Zhai et al., 2021; Fu et al., 2022; Lu et al., 2023; Wang et al., 2023). For drought treatment, the roots were immersed in Hoagland’s nutrient solution supplemented with 10% and 20% PEG-6000, and sampled at 0, 0.5, 1, 2, 3, 4, 5, 6, and 7 h after treatment. For saline-alkali treatment, the roots were immersed in Hoagland’s nutrient solution supplemented with 50 and 150 mmol/L NaCl/NaHCO_3_ and sampled at 0, 0.5, 1, 3, 6, 12, 24, 36, and 48 h after treatment. For heavy metal treatment, the roots were immersed in Hoagland’s nutrient solution supplemented with 5 μmol/L and 30 μmol/L CdCl_2_, and sampled at 0, 1, 2, 3, 4, 5, and 6 d after treatment. For cold treatment, the seedlings were grown in a light incubator at 6°C and 12°C (16/8 h light/dark), and sampled at 0, 0.5, 1, 3, 6, 12, 24, 36, and 48 h after treatment. Samples of leaf and root, that mixed from at least 3 plants, respectively, were placed in sterile 5-mL centrifuge tubes, snap-frozen in liquid nitrogen and stored at −80°C for subsequent RNA isolation.

### 2.8 RNA isolation and quantitative Real-time PCR (RT-qPCR)

Total RNA was extracted from plant materials using TRIzol reagent (Takara), in accordance with the manufacturer’s instructions. Reverse transcription was performed with 2 µg of total RNA using ToloScript ALL-in-one RT EasyMix for qPCR (Tolobio), following the manufacturer’s instructions. The *PvDi19* gene was detected by RT-qPCR in response to various stresses. The reaction system consisted of 10 μL of 2 × Q3 SYBR qPCR Master Mix (Tolobio), 2.0 μL of diluted cDNA template, 0.4 μL primers, and ddH_2_O up to 20 μL. The reaction conditions were as follows: 95°C for 30 s, followed by 40 cycles of 95°C for 10 s and 60°C for 30 s. Primers for RT-qPCR were designed against non-conserved regions of the common bean *Di19* gene using the online tools of NCBI (Supplementary Table S1). Three biological replicates and two technical tests were performed for all experiments. Relative gene expression levels were calculated according to the 2^¬ΔΔCt^ method (Livak and Schmittgen, 2002).

## 3 Results

### 3.1 Identification and characterization of *PvDi19*s

Based on BLASTP multiple sequence alignments and conserved domain analysis, by removing different transcripts of the same gene, 5 to 7 high-confidence *Di19* genes were identified in common bean, mung bean, chickpea, tepary bean, barrel medic, and rice, respectively (Supplementary Table S2). All *Di19* genes were numbered and named according to their chromosomal positions. The number of *Di19* genes appears to differ slightly between dicot and monocot species, except for a slight expansion in maize (9). Notably, this count is nearly doubled in tetraploid soybeans (15) compared to other species.

In the common bean, six members of the *Di19* gene family were identified, numbered according to their chromosomal location and named *PvDi19-1* to *PvDi19-6*. Table 1 presents detailed information regarding the protein characteristics of PvDi19s. The amino acid lengths encoded by these 6 genes range from 196 to 234 aa, with molecular weights ranging from 21,797.88 Da for PvDi19-5 to 26,260.38 Da for PvDi19-2, with their pIs ranging from 4.54 to 5.89. The hydropathicity index of all PvDi19 proteins was < zero, and the aliphatic index was <100, indicating hydrophilicity. The instability index of PvDi19s exceeded 40, indicating instability. Regarding subcellular localization, all PvDi19 proteins were found to be localized to the nucleus. These results suggest that PvDi19 proteins are involved in similar biological processes, such as the regulation of gene expression in the nucleus as a TF.

**TABLE 1:** The detail information and sequence characterization of *Di19s* in common bean.

Gene name	Gene locus ID	Number of amino acids (aa)	Theoretical pI	Molecular weight	Instability index	Aliphatic index	Grand average of hydropathicity	Subcellular localization
*PvDi19-1*	Phvul.001G210800	216	4.54	24365.80	50.28	63.61	−0.502	Nuclear
*PvDi19-2*	Phvul.002G231900	234	5.87	26260.38	60.02	79.19	−0.432	Nuclear
*PvDi19-3*	Phvul.003G205500	204	5.23	22988.59	43.75	69.36	−0.606	Nuclear
*PvDi19-4*	Phvul.007G199600	220	5.67	24432.43	59.29	74.45	−0.453	Nuclear
*PvDi19-5*	Phvul.009G173300	196	4.67	21797.88	51.63	58.67	−0.637	Nuclear
*PvDi19-6*	Phvul.009G252700	217	5.89	24207.25	63.42	78.57	−0.451	Nuclear

### 3.2 Chromosomal localization and duplication of *PvDi19* genes

**FIGURE 1:**
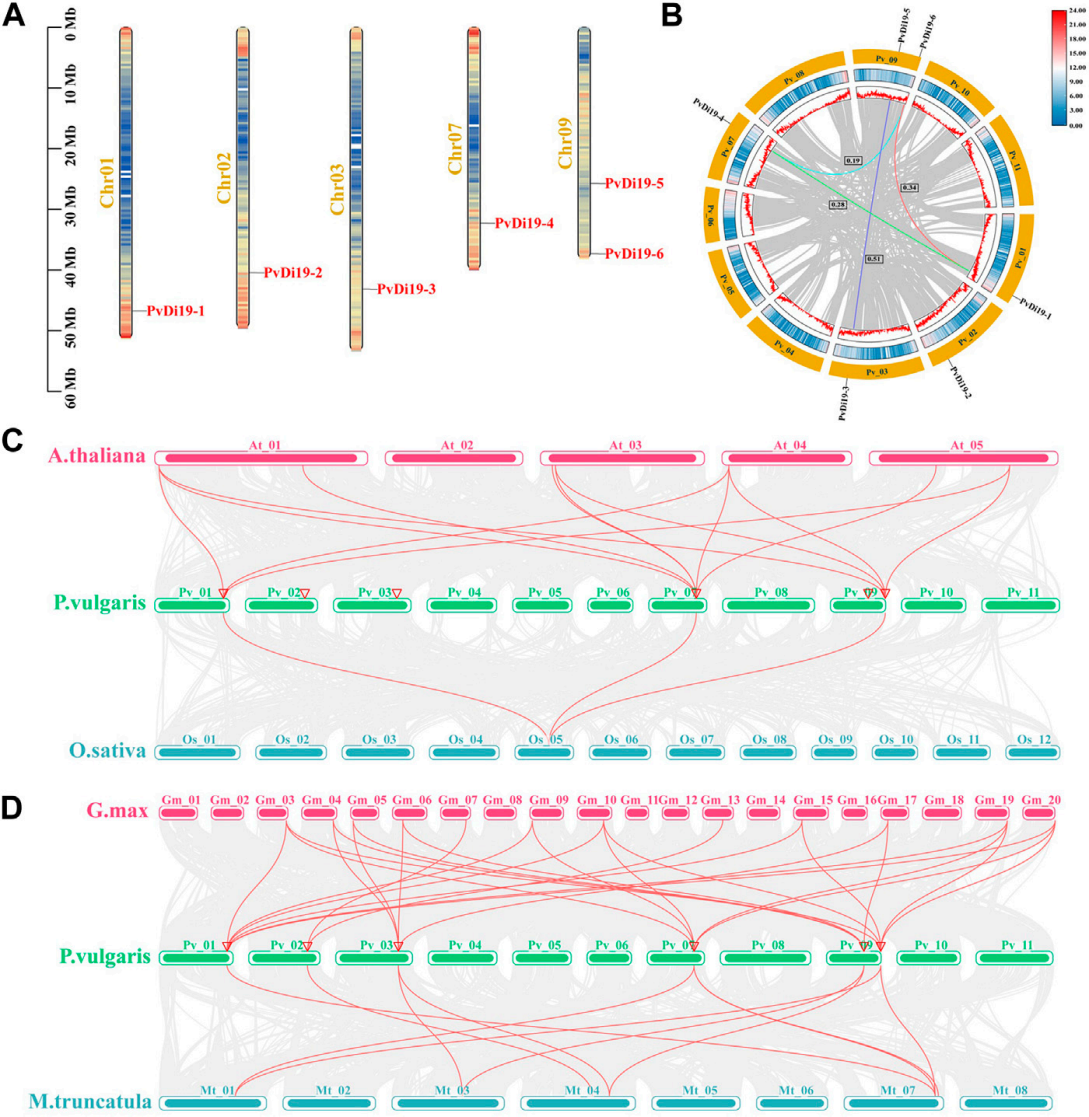
Chromosomal localization and duplication events of *PvDi19* genes. **(A)** Distribution of *Di19* genes on chromosomes of common bean. **(B)** The collinearity analysis of *Di19* genes in common bean. The outside circle colored with yellow represents different chromosomes, the middle circle represents the gene density of the chromosome (increases from blue to red). Colored lines represent the segmental duplication gene pairs, Ka/Ks values of four segmental duplication gene pairs are displayed by black boxes. **(C)** Collinearity analysis of *Di19* genes between common bean (*P.vulgaris*) with *Arabidopsis* (*A.thaliana*) and rice (*O.sativa*). **(D)** Collinearity analysis of *Di19* genes between common bean with soybean (*G.max*) and barrel medic (*M.truncatula*). The collinear gene pairs of *Di19s* in different species is showed with red lines. The chromosomes represent by curved rectangles, and red triangles on common bean chromosome represent the location of *PvDi19* genes.

As shown in Figure 1A, the physical locations of *PvDi19* genes were mapped onto 5 common bean chromosomes. *PvDi19-1* to *PvDi19-4* were located on chromosomes 1, 2, 3, and 7, respectively, whereas the remaining 2 were located on chromosome 9. Except for *PvDi19-5*, the other *PvDi19s* were found to be distributed at the terminal or sub-terminal regions of chromosomes. To further investigate duplication patterns involving *PvDi19* genes, the tandem and segmental duplication encompassing *PvDi19s* were analyzed. As shown in Figure 1B, there was no tandem duplication evident involving *PvDi19* family genes, whereas five *PvDi19s* were found to be associated with segmental duplication events, which formed four segmental duplication gene pairs. The Ka/Ks value of all segmental duplication gene pairs was <1 (Figure 1B; Supplementary Table S3), implying that *PvDi19s* were generally subject to purifying selection during evolution.

To further investigate mechanisms of amplification and evolution of *Di19*, interspecies collinearity analysis was performed between common bean and *Arabidopsis*, rice, soybean, and barrel medic. As shown in Figure 1C, *PvDi19-1*, *PvDi19-4* and *PvDi19-6* formed 13 and 3 collinear gene pairs with *AtDi19s* and *OsDi19s*, respectively. However, the more collinear gene pairs were identified in Leguminosae, and collinear genes of each member of the common bean *Di19* gene family could be found in both soybean and barrel medic (Figure 1D). These results suggest that the number of collinear gene pairs may reflect the genetic relationships of various species.

### 3.3 Phylogenetic analysis of common bean *Di19* gene family

**FIGURE 2:**
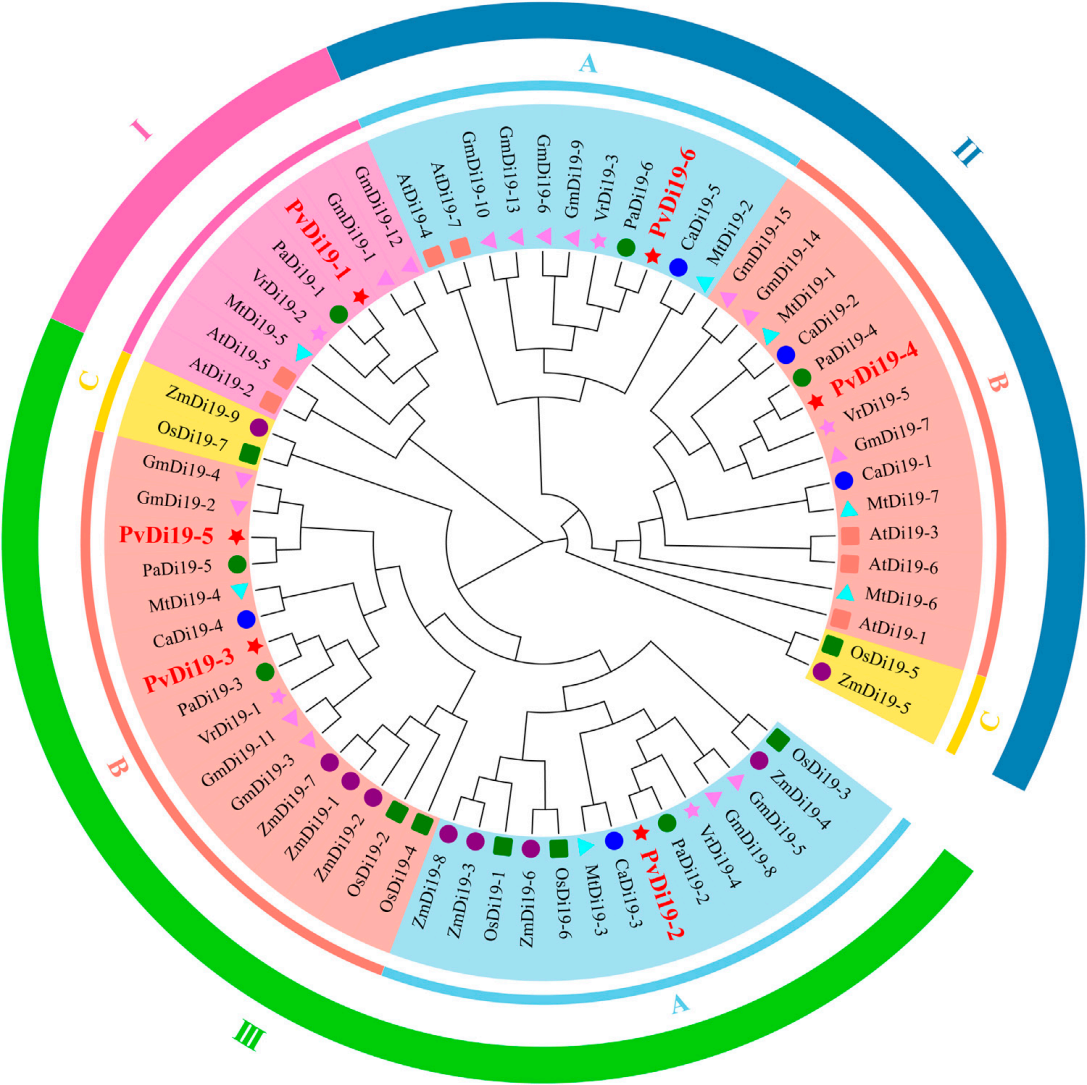
The phylogenetic tree of Di19 proteins in nine plants. A maximum likelihood (ML) phylogenetic tree is constructed using Di19 protein sequences from two monocots and seven dicots species, including *Arabidopsis *(At), common bean (Pv), soybean (Gm), mung bean (Vr), chickpea (Ca), tepary bean (Pa), barrel medic (Mt), maize (Zm), and rice (Os). I, II, III represent different evolutionary branches, A, B, C represent different sub-branches. Different shapes with different color that before the node represent different species.

To investigate phylogenetic relationships of the common bean *Di19* family, a ML phylogenetic tree was constructed using Di19 protein sequences from two monocots and seven dicots. In total, 67 Di19 proteins were divided into three evolutionary branches (Figure 2). Clade I, which included only members of the dicot species *Di19* gene family, consisted of 2 from *Arabidopsis*, 2 from soybean, and 4 from barrel medic, common bean, tepary bean, and mung bean. There were three sub-branches in Clade II, II-C included 2 genes from the monocot species rice and maize, and the remaining 2 consisted of 11 and 14 genes that originated only from dicot species. Within Clade III, 32 genes were identified from all species, with the exception of *Arabidopsis*. III-A consisted of equal numbers of genes from monocot and dicot species. III-B consisted of 5 and 11 genes from monocot and dicot species, respectively. III-C includes two genes from rice and maize. These results indicate that members of the *Di19* gene family display obvious phylogenetic differentiation in monocots and dicots.

### 3.4 Gene structure, motif composition, and protein domains of PvDi19s

**FIGURE 3:**
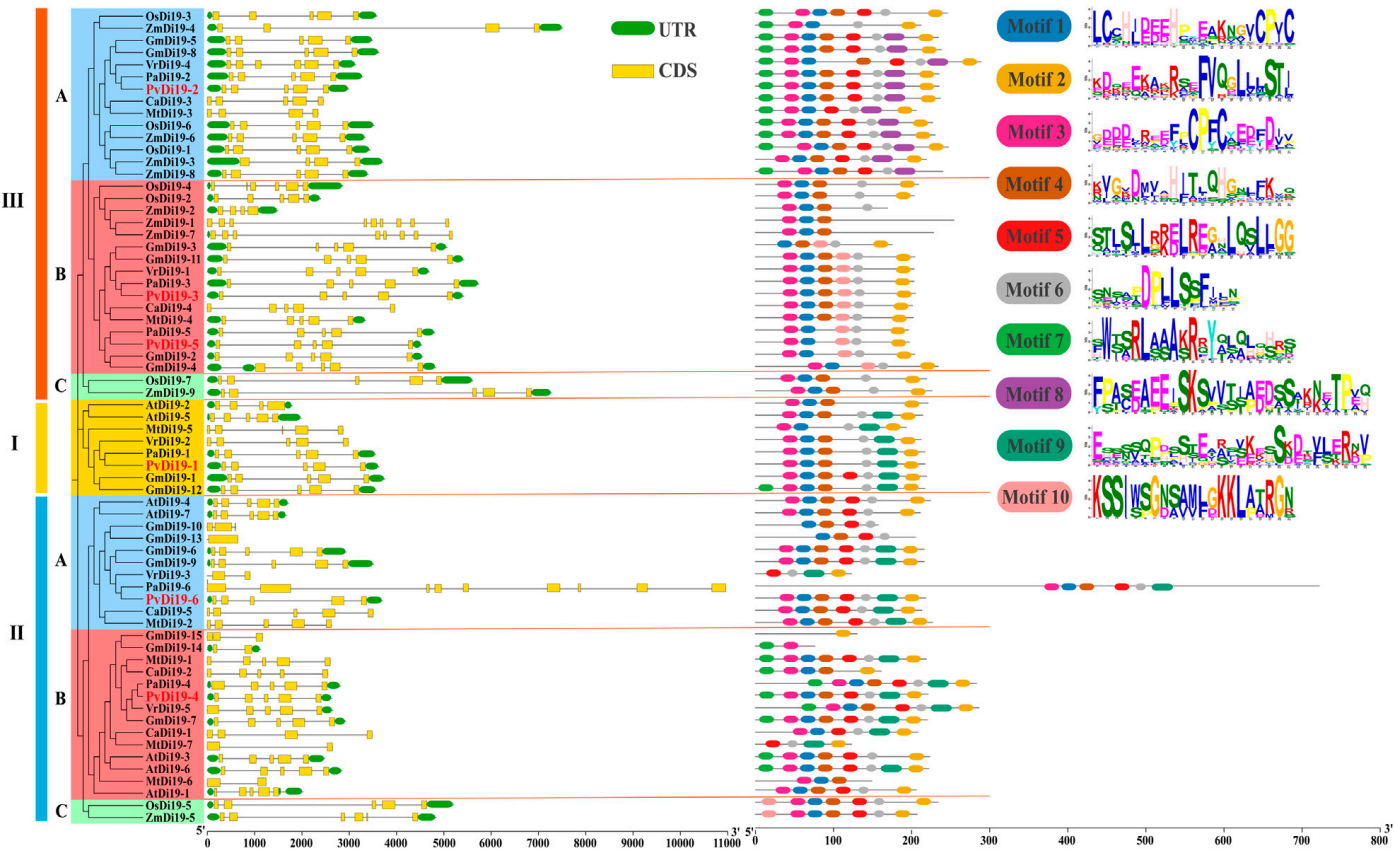
Gene structure and conserved motifs of *Di19*s in common bean. Different color backgrounds represent different subfamilies. Gene structure and conserved motifs are extracted from genome annotation information and predicted by TBtools and MEME software, respectively. The exons, UTR regions and introns are displayed by yellow boxes, green boxes and the block lines, respectively.

Differences in the structure of *Di19* genes were compared based on structural annotation information of reference genomes from various species (Figure 3). Overall, the *Di19* gene structure was relatively conserved, with most having similar exons and introns. However, a certain degree of diversity in gene structure was observed in some *Di19s*, such as *MtDi19-6*, *MtDi19-7*, *GmDi19-14* and *VrDi19-3* which contained only two exons, whereas *ZmDi19-1*, *ZmDi19-7* and *PaDi19-6* contained a greater number of exons. In common bean, all *PvDi19s* contained five exons and possessed similar gene structures, although they originated from different evolutionary branches.

We also analyzed the composition and arrangement of Di19 conserved motifs from nine species based on the MEME program using ten motifs (Figure 3). The results showed that motifs 1, 2, 3, 4, and 6 were conserved in almost all Di19 proteins from various species, and these five motifs were identified in all common bean Di19 proteins, except for PvDi19-5. However, some motifs were present in certain evolutionary branches. For example, motif 9 was present only in Clades I and II, and motif 8 was present only in Clade III-A. It revealed that the most closely orthologous genes in terms of phylogenesis exhibited similar composition and arrangement of conserved motifs, such as PvDi19 and PaDi19 proteins. This suggests that the identified conserved motifs are highly consistent with their phylogenetic relationships, but are, however, not related to species specificity.

**FIGURE 4:**
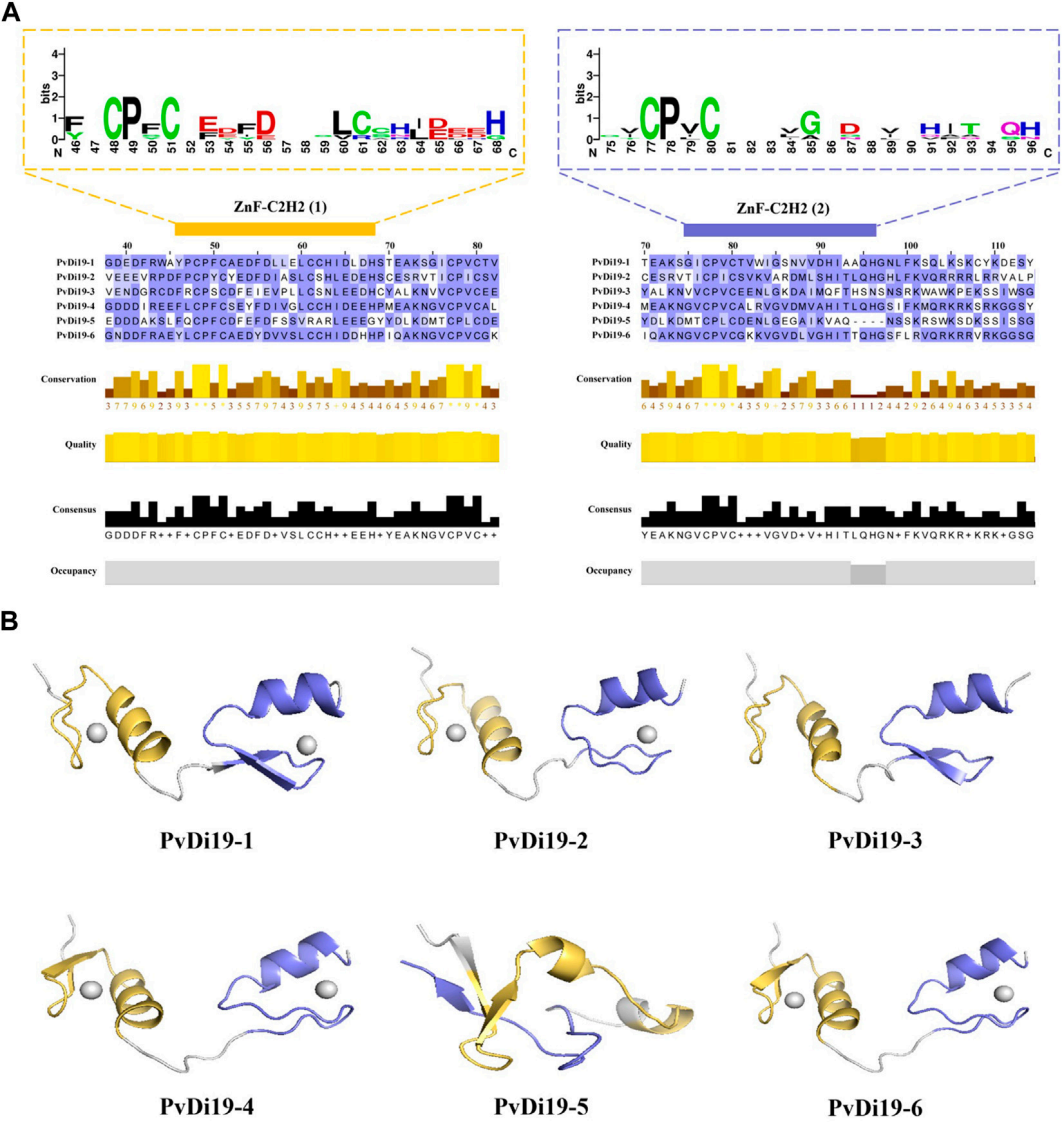
Multiple sequence alignment and tertiary structure prediction of PvDi19 protein. **(A)** Multiple sequence alignment of two C2H2-type zinc finger structural domains (ZnF-C2H2) of PvDi19 protein. Amino acid sequence of two ZnF-C2H2 domains are shown in two dashed boxes that colored with yellow and purple, the larger the letter, the more conserved the amino acid. **(B)** Tertiary structure prediction of ZnF-C2H2 domain. The tertiary structure of two ZnF-C2H2 domain colored with yellow and purple, respectively.

Furthermore, multiple sequence alignments of PvDi19 proteins were performed using Clustal Omega and protein domains were visualized using Jalview software. As shown in Figure 4A, the spacing of the zinc-binding ligands in the first and second zinc-finger domains was 11 and 10 amino acid residues, respectively (amino acids 52–62, 81–90). There were 2 spacings of 2 cysteine residues and 4 spacings of 2 histidine residues in both zinc finger domains. The cysteine residues were highly conserved in both ZnF-C2H2 motifs, whereas the major variation was present in histidine residues in PvDi19-3 and PvDi19-5. In PvDi19-3, histidine residues (H) were replaced with asparagine (N), glutamine (Q), or asparagine (N) at aa positions 63, 91, and 96, respectively. In PvDi19-5, three H residues were replaced with arginine (R), glycine (G), and valine (V) at aa positions 63, 68, and 91, respectively.

To further investigate the effect of these variations on the tertiary structure of PvDi19, the protein tertiary structure predictions were performed using SWISS-MODEL and were visualized using PyMOL software. As shown in Figure 4B, PvDi19-5 presented an atypical tertiary structure that differed from those of the other members of the PvDi19 family. Although PvDi19-3 presented a tertiary structure similar to that of other PvDi19s, the zinc ions that bind to it were not predicted, probably because of variations involving histidine residues.

### 3.5 Phosphorylation site prediction of PvDi19 protein

**FIGURE 5:**
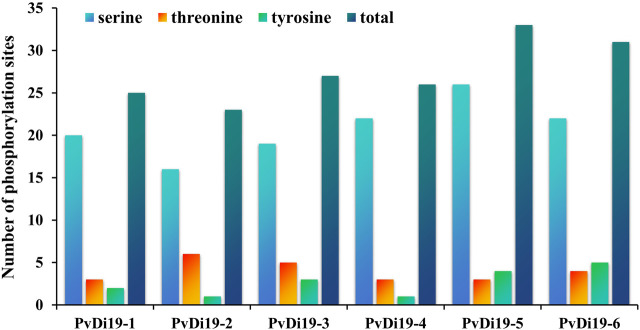
Analysis of phosphorylation sites (serine, threonine, and tyrosine) in PvDi19 proteins.

Phosphorylation, one of the most basic and universal protein post-translational regulatory mechanisms, has important effects on protein function. In this work, the NetPhos 3.1 server was used to predict the phosphorylation sites of PvDi19 proteins. The results (Supplementary Table S4) revealed that common bean Di19 proteins harbor many phosphorylation sites for protein kinases (PK). The phosphorylation sites of PKA, PKC, and CKII were predicted for all the common bean Di19 proteins, among which the number of PKC phosphorylation sites was the largest. Based on these results, the number of three kind of phosphorylation sites (serine, threonine, and tyrosine) was compared in PvDi19 proteins. As shown in Figure 5, most family members had more serine phosphorylation sites and fewer tyrosine phosphorylation sites. In addition, PvDi19-4 and PvDi19-6 had more potential phosphorylation sites than other members.

### 3.6 Cis-acting elements and spatiotemporal expression patterns of *PvDi19s*

**FIGURE 6:**
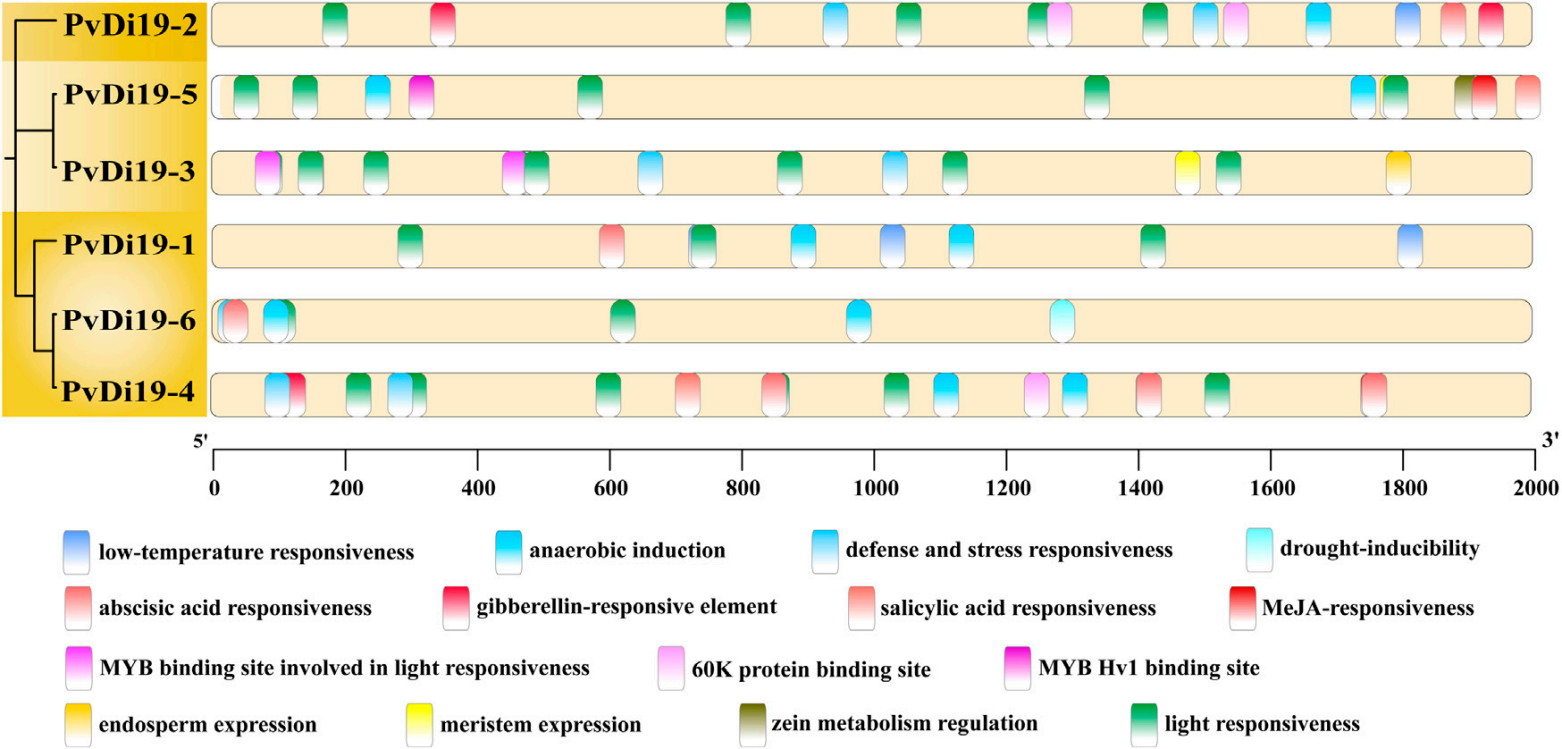
The cis-acting elements in the promoter regions of PvDi19 genes. Different colored curved rectangles represent different types of cis-acting elements. All elements are predicted by TBtools software and modified using Adobe illustrator.

Cis-acting elements in the promoter regions of the six *PvDi19s* loci were predicted and analyzed using the PlantCare database. As shown in Figure 6, with the exception of the light-responsive element, a large number of plant hormone-related elements in the promoter region, such as abscisic acid, gibberellin, MeJA, and salicylic acid-related elements, indicate that *Di19s* may be influenced by various growth regulators. Furthermore, cis-acting elements of *PvDi19s* involved in low-temperature, drought-inducibility, defence, and stress responsiveness were identified, indicating that Di19 proteins play critical roles in the response to various stresses. The presence of several MYB and 60 K protein-binding sites partially explains their involvement in interactions with other proteins. Additionally, elements related to endosperm and meristem expression imply that Di19 proteins may be involved in various plant growth and developmental processes.

**FIGURE 7:**
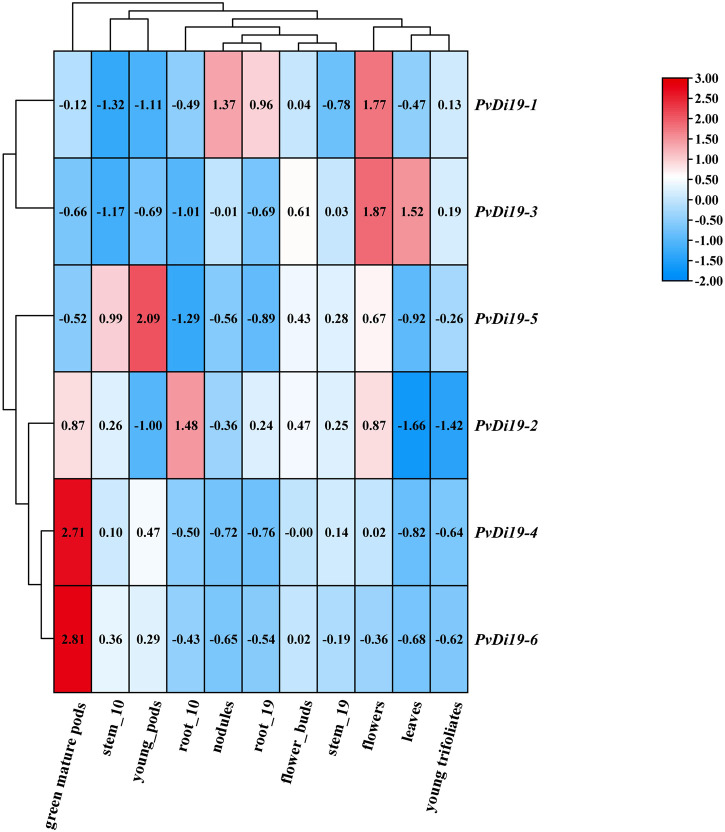
Heatmap of gene expression patterns of *PvDi19s*. All expression data are obtained from Phytozome database. The log_2_ values labeled in color bar are used to display the gene expression levels, blue and red representing at low and high transcription abundance, respectively.

To investigate the temporal and spatial expression patterns of *PvDi19s*, the gene expression data were obtained from the Phytozome server. As shown in Figure 7, expression levels of members of the *PvDi19* family exhibited relatively large differences in various and specific tissues across multiple developmental stages. Specifically, almost all *PvDi19s* had lower expression levels in young trifoliates and leaves, with the exception of *PvDi19-3*; *PvDi19-4* and *PvDi19-6,* which were highly expressed in green mature pods, while *PvDi19-5* was highly expressed in young pods. Expression levels of *PvDi19-1* and *PvDi19-3* were highest in flowers, and *PvDi19-1* and *PvDi19-2* were highest in nodules and roots, respectively.

### 3.7 Response of *PvDi19s* to various abiotic stresses

**FIGURE 8:**
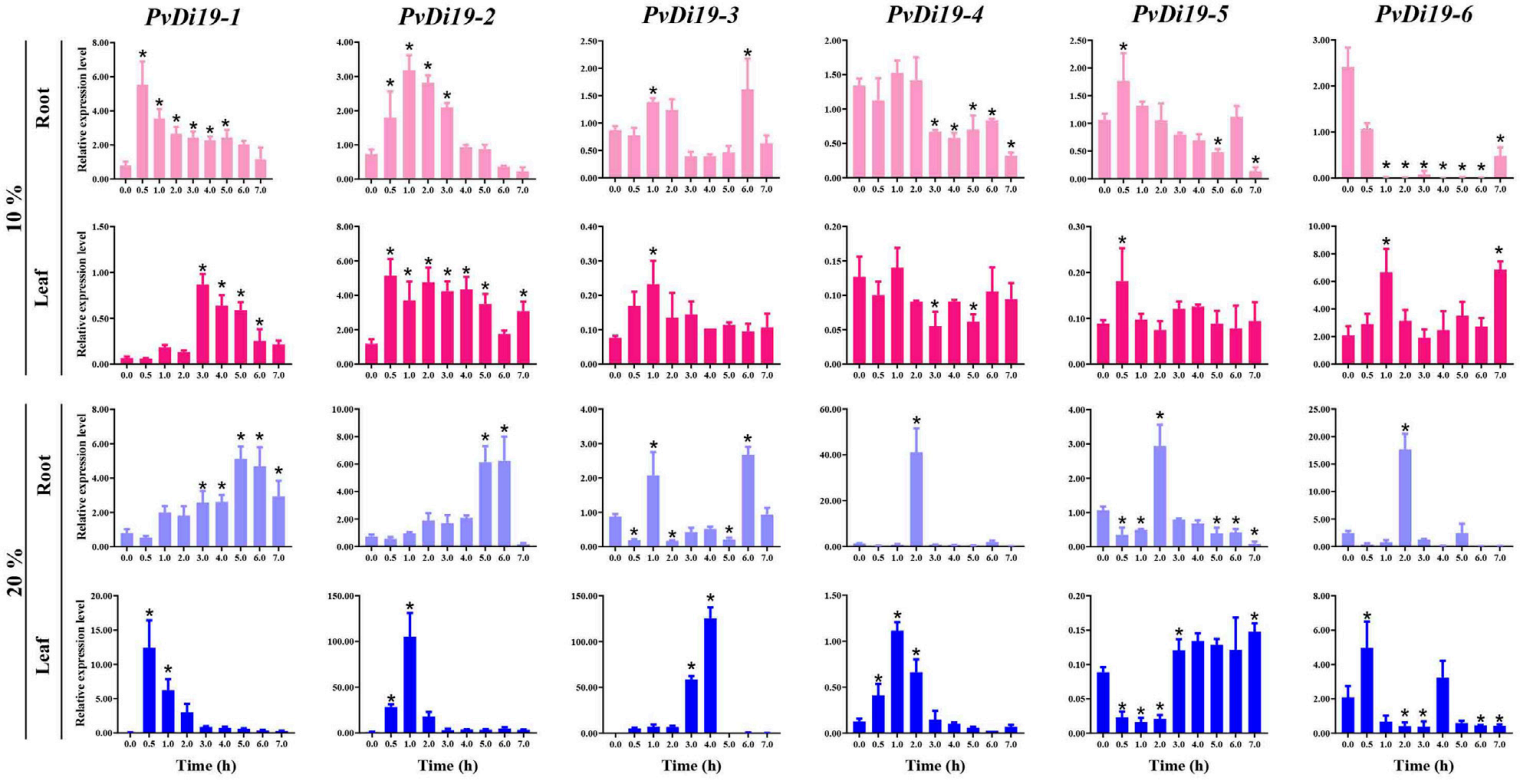
Expression patterns of *PvDi19s* under PEG treatment. The error bars indicate standard deviation (SD) (n=3). Significance analysis is performed between the data of pre-treatment (0 h or 0 d) with each time point after treatment. *, *p* < 0.01.

To investigate the roles of common bean *PvDi19* family genes in defense against abiotic stresses, RT-qPCR was used to examine gene expression in response to continuous osmotic, saline, alkaline, heavy metal, and cold stresses. As shown in Figure 8 and Supplementary Figures S1-S4, the dynamic expression patterns of these genes exhibited relatively large differences in response to various abiotic stressors. For example, after 10% PEG treatment (Figure 8), the expression levels of *PvDi19-1*, *PvDi19-2* and *PvDi19-5* in roots increased to a maximum at 0.5 h, 1 h and 0.5 h, respectively, then decreased gradually with treatment time. Expression of *PvDi19-3* in leaves showed a similar trend. The expression levels of *PvDi19-3* and *PvDi19-4* in roots were significantly inhibited after 3 h of treatment, whereas *PvDi19-6* was inhibited at all time points. In leaves, the expression of *PvDi19-1* was significantly upregulated after 3 h of treatment, whereas *PvDi19-2* was always activated during continuous stress. When suffering severe osmotic stress (20% PEG) (Figure 8), expression of *PvDi19-1* and *PvDi19-2* in roots was gradually upregulated after 1 h of treatment. The remaining genes in the roots, including *PvDi19-3*, *PvDi19-4*, *PvDi19-5*, and *PvDi19-6* were inhibited during continuous stress, except at certain detected time points. This expression profile was consistent with the variation in *PvDi19-3* and *PvDi19-6* in leaves. Interestingly, *PvDi19-1*, *PvDi19-2,* and *PvDi19-4* in leaves were significantly induced from 0.5 to 2 h of treatment, whereas *PvDi19-5* was inhibited during this period.

As revealed by RT-qPCR, at lower concentrations (50 mmol/L) of salt stress (Supplementary Figure S1), the expression of genes, including *PvDi19-1* and *PvDi19-2* in roots, and *PvDi19-2* and *PvDi19-3* in leaves, exhibited a variation in the unimodal curve. The expression levels of *PvDi19-3* and *PvDi19-6* in roots, as well as *PvDi19-4* and *PvDi19-5* in leaves, were rapidly upregulated in the final stage of treatment. The expression levels of *PvDi19-6* and *PvDi19-1* in leaves were significantly upregulated at the early and medium stages, respectively. At higher salt concentrations (150 mmol/L) (Supplementary Figure S1), the expression levels of *PvDi19-1* in roots increased after 1 h of stress, peaked at 6 h of stress, and then decreased gradually. The expression of *PvDi19-2* in roots was significantly increased from 1 to 36 h of treatment. Similarly, the expression of *PvDi19-5* in leaves was significantly induced from 3 to 24 h of treatment. In addition, the expression levels of *PvDi19-6* in roots and those of *PvDi19-1*, *PvDi19-2*, *PvDi19-3,* and *PvDi19-6* in leaves were significantly upregulated after 12 h of treatment.

Under lower concentrations (50 mmol/L) of alkaline treatment (Supplementary Figure S2), the expression of *PvDi19-2* in roots was upregulated from 0.5 to 3 h and subsequently returned to normal levels. With the exception of induction at 0.5 h, the expression of *PvDi19-3* in roots was inhibited at all timepoints. Interestingly, the expression of *PvDi19-4* and *PvDi19-5* decreased gradually in roots but was significantly upregulated in leaves after 6 h of treatment. Similar to the variation trend observed for *PvDi19-6* in roots, the expression of *PvDi19-1*, *PvDi19-2*, and *PvDi19-6* in leaves was also significantly upregulated after 12 h of treatment. When subjected to a higher concentration (150 mmol/L) of alkaline treatment (Supplementary Figure S2), the expression of *PvDi19-1* and *PvDi19-2* in roots presented a variation trend in the unimodal curve and increased to a maximum level after 3 h of treatment. In roots, *PvDi19-3* and *PvDi19-4* were significantly repressed during continuous stress. *PvDi19-5* expression was inhibited after 24 h of treatment. In contrast, *PvDi19-6* expression significantly increased during this period. In leaves, the expression of *PvDi19s* exhibited a consistent variation tendency; it was inhibited during continuous stress, except at some limited tested time points.

When subjected to lower concentrations of Cd stress (5 μmol/L) (Supplementary Figure S3), the expression of *PvDi19-1*, *PvDi19-2*, *PvDi19-3*, and *PvDi19-4* in roots was significantly upregulated on day 6 of treatment. However, the expression of these genes was significantly inhibited in leaves. Moreover, most *PvDi19* genes in the leaves were inhibited at higher concentrations of Cd (30 μmol/L), except for *PvDi19-5* (Supplementary Figure S3). In roots, the expression levels of *PvDi19-2*, *PvDi19-3*, and *PvDi19-5* decreased gradually when subjected to higher concentrations of Cd (30 mmol), whereas the expression of *PvDi19-4* and *PvDi19-6* was upregulated in the early and final stages of treatment.

Furthermore, expression of *PvDi19-6* in roots was upregulated significantly from 0.5 to 3 h under moderate cold treatment (12°C), whereas the other genes were induced at the medium stage of treatment (Supplementary Figure S4). In leaves, expression of *PvDi19* family genes was inhibited under moderate cold treatment (12°C), with the exception of induced expression at limited detected time points. Additionally, these genes shared similar expression patterns when undergoing severe cold treatment (6°C) (Supplementary Figure S4), and most were induced rapidly in response to stressors and then were subsequently inhibited. Distinctively, expression levels of *PvDi19-3*, *PvDi19-4*, and *PvDi19-5* in roots increased gradually and peaked at 3–6 h under severe cold treatment (6°C). The above results indicate that different *PvDi19* genes were alternately upregulated in different tissues and times and functioned in response to cold stress.

## 4 Discussion

### 4.1 Characteristics of common bean *Di19* gene family members

In plants, the Di19 family usually comprises several members encoded by a small family of genes (Feng et al., 2015). Seven members of the *Di19* family have been identified in *Arabidopsis* (Milla et al., 2006), rice (Wang et al., 2016), and soybean (Feng et al., 2015), respectively. Moreover, 10 and 8 Di19 proteins have been identified in moso bamboo (*Phyllostachys edulis*) and poplar (*Populus L.*), respectively (Wu, 2018; Wu, 2022). Notably, a greater number of *Di19* genes have been identified in hexaploid wheat (18) (Du et al., 2023), probably because of its polyploidy status. However, there have been no reports referring to common bean *Di19* gene family members. Thus, the identification of *Di19* genes in common bean, as well as bioinformatics and gene expression analyses, were performed in this study to predict the roles of these genes in abiotic stress.

In this study, approximately 5–9 putative *Di19* genes were identified in common bean (7), chickpeas (5), mung beans (5), tepary bean (6), barrel medic (7), and some monocotyledons maize (9) and rice (7). However, the number of gene members of the *Di19* family is nearly doubled in tetraploid soybeans (15). It has been confirmed that alongside polyploidy or whole-genome duplication, segmental and tandem duplications are the main pathways of gene family expansion (Maher et al., 2006). Some gene families contain hundreds of members, usually because numerous family genes are involved in tandem and segmental duplications (Yang et al., 2021; Li et al., 2022). The results of collinearity analysis revealed (Figure 1B) that only four segmental duplication gene pairs involving common bean *Di19* family members (*PvDi19-1* and *PvDi19-4*; *PvDi19-1* and *PvDi19-6*; *PvDi19-4* and *PvDi19-6*; *PvDi19-3* and *PvDi19-5*), which differed from some gene families which contained both tandem and segmental duplication blocks. Moreover, only three *PvDi19* genes, *PvDi19-1, PvDi19-4* and *PvDi19-6,* which are located on chromosomes 1, 7, and 9, respectively, formed synteny blocks in *Arabidopsis* and rice, and the number of collinear genes between common bean and *Arabidopsis* was greater than that in rice (Figure 1C). In addition, the collinear genes of all common bean *Di19* genes were identified in soybean and barrel medic (Figure 1D), although not all Ka/Ks values were calculated because of high sequence divergence, suggesting that the closer the genetic relationship and phylogenesis, the greater the number of collinear genes. These results are in according with those of previous studies and demonstrate that segmental duplication is the predominant means of expansion in the *Di19* gene family in common bean and other species (Du et al., 2023).

Furthermore, several approaches were used to investigate the similarities between PvDi19 proteins. First, a phylogenetic analysis was performed on the common bean and 8 other species, including monocotyledons and dicotyledons. A phylogenetic tree was constructed using the complete amino acid sequences of these proteins. In different distinct evolutionary branches, Clade I, Clade II-A, and Clade II-B only contained dicot *Di19s*, Clade II-C and Clade III-C only contained the genes from monocot rice and maize. Similar results have been reported in previous studies of different species (Milla et al., 2006; Du et al., 2023), indicating the diversity of Di19 proteins in monocots and dicots. However, some Di19 proteins from both monocots and dicots was distinguished into the same sub-branches (III-A and III-B), suggesting that these *Di19* genes might have arisen from recent gene duplication events (Milla et al., 2006). Secondly, gene structures and motif compositions were compared for *Di19* genes from various species. Overall, *Di19s* exhibited a conserved exon-intron structure, although certain genes, such as *AtDi19-2*, *MtDi19-6/7*, *GmDi19-14*, *VrDi19-3*, *ZmDi19-1/7* and *PaDi19-6*, contained different numbers of exons than the 5 found in most genes. Meanwile, *Di19* genes in the same sub-branch shared similar lengths of genomic sequences, indicating the conservation of *Di19s* during evolution. The most closely orthologous genes in terms of phylogenesis exhibited similar composition and arrangement of conserved motifs, indicating that the composition of conserved motifs showed no species specificity but was highly consistent with their phylogenetic relationships.

We also performed multiple sequence alignments of PvDi19 proteins. In general, the Di19 protein contains a highly conserved zf-Di19 domain close to the N-terminus (Gosti et al., 1995; Milla et al., 2006), which consists of two C2H2 ZFP motifs and is a characteristic DNA-binding motif in eukaryotic TFs. The amino acid sequences of the two ZFP motifs are represented by the canonical Cys–X_2,4_–Cys–X_12_–His–X_3,4,5_–His with slight differences (Brown et al., 1985; Liu et al., 2013). In *Arabidopsis*, the putative C2H2 zinc-finger motifs, as well as the spacing between them, are strictly conserved, except for AtDi19-5 (Miller et al., 1985). In the present study, the cysteine residues of the two ZnF-C2H2 motifs were highly conserved in the common bean Di19 protein, whereas major variation was present involving histidine residues in PvDi19-3 and PvDi19-5. In PvDi19-5, three histidine residues were replaced with arginine, glycine, and valine at positions 63, 68, and 91, respectively. These variations are responsible for the diversity in the tertiary structure of the PvDi19 protein (Figure 4B). It has been shown that the tertiary structures of PvDi19-5 are very different to other Di19 proteins.

### 4.2 Functional divergence of common bean *Di19* genes

It has been confirmed that the Di19 protein functions as a TF, regulating the expression of related genes in response to various abiotic stresses (Milla et al., 2006; Li G. et al., 2010; Li S. et al., 2010; Liu et al., 2013; Qin et al., 2014; Feng et al., 2015; Wang et al., 2016). To explore the functions of *Di19* family members in common bean, cis-regulatory elements of *PvDi19s* were predicted using the PlantCare database, firstly. It is well known that the analysis of cis-regulatory elements is a universal approach for predicting the putative function of gene families using bioinformatics (Zhou et al., 2024). In this study, various cis-elements in the promoter regions of common bean *Di19* genes involved in hormone responses, stress responses, and endosperm and meristem development, implying a potential role for common bean *Di19* genes. Several MYB-binding sites were also detected in *PvDi19-2*, *PvDi19-3*, and *PvDi19-5*. It has been confirmed that MYB proteins, which are critical TFs, are involved in responses to abiotic stress in plants (Alexander et al., 2019), and the overexpression of some *MYB* genes in *Arabidopsis* and tobacco enhances abiotic stress tolerance in transgenic plants (Zhang et al., 2014; Zhao J. et al., 2017; Wei et al., 2017). In cotton, at least 3 and 8 MYC recognition sites have been identified in the promoter regions of *GhDi19-1* and *GhDi19-2*, respectively (Li G. et al., 2010). These TF recognition and binding sites are also present in the promoter regions of several dehydration-responsive genes (such as *AtMYB2* and *AtMYC2*), which encode known transcriptional activators of ABA signaling (Abe et al., 2003). These results indicated that these 3 *PvDi19s* probably function as transcriptional activators of ABA-inducible gene expression under drought stress in common bean.

To further investigate the roles of *Di19* genes in common bean in response to various stresses, the relative expression levels of genes were analyzed by RT-qPCR. Overall, the expression patterns of *PvDi19* genes were diverse in different tissues and under different degrees of abiotic stress. Specifically, inhibition of *PvDi19s* was more pronounced under severe PEG stress (20% PEG). *PvDi19-1* and *PvDi19-2* in roots exhibited greater hypersensitivity in response to 10% PEG treatment, and in leaves, showed greater hypersensitivity in response to 20% PEG treatment. Thus, it speculated that *PvDi19-1* and *PvDi19-2* may play critical roles in responses to drought stress. It reported that *AtDi19-1* and *AtDi19-3* were rapidly induced by dehydration in *Arabidopsis* (Milla et al., 2006), *GmDi19-5*, *GmDi19-6*, and *GmDi19-3* were upregulated by > 5-fold after 5 h of drought stress (Feng et al., 2015). In wheat, *TaDi19-6* in roots and *TaDi19-5* and *TaDi19-7* in shoots were significantly upregulated after drought stress (Du et al., 2023). In addition, most *PvDi19* genes were insensitive to saline-alkali stress, some of which increased gradually and peaked at the intermediate stage of treatment, whereas others were inhibited or not regulated by saline-alkali stress at an early stage. Notably, the expression of *PvDi19-6* was significantly upregulated at the late stage of treatment, indicating its special role in saline-alkali stress. Milla et al. (2006) reported that *AtDi19-2* and *AtDi19-4* are upregulated in response to high salt stress in *Arabidopsis*. Most soybean *Di19* genes were induced by salt treatment, and transcripts of *GmDi19-5* increased >10-fold after 12 h (Feng et al., 2015). The upregulation of *TaDi19–5* and *TaDi19–6* was >10-times higher in shoots and seedling roots after salt treatment in wheat, respectively (Du et al., 2023). Moreover, expression of the most common bean *Di19* gene was inhibited or not regulated by different degrees of Cd stress at the most detected time. However, the expression of *PvDi19-6* in roots was significantly upregulated at lower concentrations of cadmium (5 mmol/L), indicating a specific role in the response to heavy metal stress. Analysis of gene expression under cold treatment indicated that common bean *Di19* genes exhibited greater sensitivity to severe cold stress (6°C) and expression of most genes, both in roots and leaves, was upregulated rapidly at early stages (0.5 h). However, when suffering moderate cold treatment (12°C), expression of most *PvDi19* genes was upregulated significantly at limited detected time points, which probably relate to its greater cold resistance obtained during long-term evolution. All above results provide clear evidence for the functional divergence of *Di19s* in different species under various stresses. However, Furthermore, numerous transgenic *Arabidopsis* plants were generated to verify the roles of *Di19* genes in various stress signaling pathways. For example, overexpression of *ZmDi19-1* in *Arabidopsis* enhances salt tolerance in transgenic plants (Zhang et al., 2019), whereas overexpression of *TaDi19-7* in *Arabidopsis* attenuates salt and osmotic stress tolerance in transgenic plants (Du et al., 2023). Additionally, the *OsDi19-4*-overexpressing lines showed greater hypersensitivity than *OsDi19-4*-knock-down transgenic lines in response to ABA treatment during seed germination and post-germination seedling growth (Wang et al., 2016), and *ZmDi19-1* transgenic *Arabidopsis* exhibit decreased sensitivity to ABA in seedlings, with less impaired and longer roots than those of WT plants (Zhang et al., 2019). In this work, several important *Di19* genes, such as *PvDi19-1*, *PvDi19-2*, and *PvDi19-6* of common bean were identified, which may play important roles in responding to abiotic stress by RT-qPCR analysis. Future genetic transformation experiments that using transgenic *Arabidopsis* plants are needed to further clarify the function of these intriguing genes.

## 5 Conclusion

The present study identified 6 *Di19* genes in common bean. These genes shared a conserved exon–intron structure, and were distinguished into three different evolutionary branches. The composition and arrangement of their conserved motifs were highly consistent with their phylogenetic relationships. PvDi19-5 presented an atypical tertiary structure owing to amino acid sequence variations in the C2H2-type zinc-finger structural domains. *PvDi19-1* and *PvDi19-2* probably play critical roles in responses to drought stress, *PvDi19-6* likely functions in saline-alkali and heavy metal stress responses. The findings of this study will be helpful for deepening the functional analysis of *PvDi19s* in responses to abiotic stress in the future and will contribute to the application of the *Di19* gene in the improvement of resistance in common bean.

## Data Availability

The original contributions presented in the study are included in the article/Supplementary Material, further inquiries can be directed to the corresponding author.
