# Supplementary material for: Identification and expression of the Di19 gene family in response to abiotic stress in common bean (Phaseolus vulgaris L.)
Source: Front Genet. 2024 May 30;15:1401011. doi: 10.3389/fgene.2024.1401011 (PMC11169598; doi:10.3389/fgene.2024.1401011)
Supplement: Supplementary file 1 [file Table1.pdf]

TABLE S1 Primer sequences for RT-qPCR.

Gene	Forward Primer	Reverse Primer
PvDi19-1	5'- ATGCCCTTTTTGCGCTGAAG-3'	5'- AGTTCCCATGTTGTGCTGCT-3'
PvDi19-2	5'-CCGCTAAACGCCAATACACG-3'	5'- TGAGAGCACAAAGACGCGAT-3'
PvDi19-3	5'- GTGGAAAATGATGGCCGGTG-3'	5'- TTCACACACGGGACAAACCA-3'
PvDi19-4	5'- CTCTAGGCGCTACCAATCCG-3'	5'- ACTCGGAACAGAATGGGCAG-3'
PvDi19-5	5'- ATCTCCAACTCTTCACGCCG-3'	5'-ACAGGACACACCCCGTTTTT-3'
PvDi19-6	5'- TTCGACTTCAGGGCTTCCAC-3'	5'- CTACGGAATCACCCGACGAG-3'
actin	5'- GAAGTTCTCTTCCAACCATCC-3'	5'- TTTCCTTGCTCATTCTGTCCG-3'

TABLE S2 The detailed list of *Di19* genes in various species.

Species	Gene name	Gene locus ID
Arabidopsis (*Arabidopsis thaliana*)	*AtDi19-1*	At1g56280
Arabidopsis (*Arabidopsis thaliana*)	*AtDi19-2*	At1g02750
Arabidopsis (*Arabidopsis thaliana*)	*AtDi19-3*	At3g05700
Arabidopsis (*Arabidopsis thaliana*)	*AtDi19-4*	At3g06760
Arabidopsis (*Arabidopsis thaliana*)	*AtDi19-5*	At4g02200
Arabidopsis (*Arabidopsis thaliana*)	*AtDi19-6*	At5g26990
Arabidopsis (*Arabidopsis thaliana*)	*AtDi19-7*	At5g49230
Barrel medic (*Medicago truncatula*)	*MtDi19-1*	Medtr1g083780
Barrel medic (*Medicago truncatula*)	*MtDi19-2*	Medtr2g035070
Barrel medic (*Medicago truncatula*)	*MtDi19-3*	Medtr4g088430
Barrel medic (*Medicago truncatula*)	*MtDi19-4*	Medtr4g104770
Barrel medic (*Medicago truncatula*)	*MtDi19-5*	Medtr7g109145
Barrel medic (*Medicago truncatula*)	*MtDi19-6*	Medtr7g112400
Barrel medic (*Medicago truncatula*)	*MtDi19-7*	Medtr7g112403
Chickpea (*Cicer arietinum*)	*CaDi19-1*	Ca_01117
Chickpea (*Cicer arietinum*)	*CaDi19-2*	Ca_03643
Chickpea (*Cicer arietinum*)	*CaDi19-3*	Ca_06337
Chickpea (*Cicer arietinum*)	*CaDi19-4*	Ca_08809
Chickpea (*Cicer arietinum*)	*CaDi19-5*	Ca_14779
Tepary bean (*Phaseolus acutifolis*)	*PaDi19-1*	Phacu.CVR.001G257900
Tepary bean (*Phaseolus acutifolis*)	*PaDi19-2*	Phacu.CVR.002G265900
Tepary bean (*Phaseolus acutifolis*)	*PaDi19-3*	Phacu.CVR.003G246100
Tepary bean (*Phaseolus acutifolis*)	*PaDi19-4*	Phacu.CVR.007G238800
Tepary bean (*Phaseolus acutifolis*)	*PaDi19-5*	Phacu.CVR.009G213800
Tepary bean (*Phaseolus acutifolis*)	*PaDi19-6*	Phacu.CVR.009G309800
Mung bean (*Vigna radiata*)	*VrDi19-1*	Vradi0223s00030

Mung bean (*Vigna radiata*)	*VrDi19-2*	Vradi03g06290
Mung bean (*Vigna radiata*)	*VrDi19-3*	Vradi05g00650
Mung bean (*Vigna radiata*)	*VrDi19-4*	Vradi07g16580
Mung bean (*Vigna radiata*)	*VrDi19-5*	Vradi08g12130
Soybean (*Glycine max*)	*GmDi19-1*	Glyma.03G217300
Soybean (*Glycine max*)	*GmDi19-2*	Glyma.04G188600
Soybean (*Glycine max*)	*GmDi19-3*	Glyma.05G041800
Soybean (*Glycine max*)	*GmDi19-4*	Glyma.06G177000
Soybean (*Glycine max*)	*GmDi19-5*	Glyma.07G200800
Soybean (*Glycine max*)	*GmDi19-6*	Glyma.09G040200
Soybean (*Glycine max*)	*GmDi19-7*	Glyma.10G149200
Soybean (*Glycine max*)	*GmDi19-8*	Glyma.13G175600
Soybean (*Glycine max*)	*GmDi19-9*	Glyma.15G145400
Soybean (*Glycine max*)	*GmDi19-10*	Glyma.16G103600
Soybean (*Glycine max*)	*GmDi19-11*	Glyma.17G124400
Soybean (*Glycine max*)	*GmDi19-12*	Glyma.19G213900
Soybean (*Glycine max*)	*GmDi19-13*	Glyma.20G104400
Soybean (*Glycine max*)	*GmDi19-14*	Glyma.20G239000
Soybean (*Glycine max*)	*GmDi19-15*	Glyma.20G238900
Maize (*Zea mays*)	*ZmDi19-1*	Zm00001eb130380
Maize (*Zea mays*)	*ZmDi19-2*	Zm00001eb139880
Maize (*Zea mays*)	*ZmDi19-3*	Zm00001eb157160
Maize (*Zea mays*)	*ZmDi19-4*	Zm00001eb240660
Maize (*Zea mays*)	*ZmDi19-5*	Zm00001eb285900
Maize (*Zea mays*)	*ZmDi19-6*	Zm00001eb295750
Maize (*Zea mays*)	*ZmDi19-7*	Zm00001eb345470
Maize (*Zea mays*)	*ZmDi19-8*	Zm00001eb360010
Maize (*Zea mays*)	*ZmDi19-9*	Zm00001eb410020

Rice (*Oryza sativa*)	*OsDi19-1*	LOC_Os01g48190
Rice (*Oryza sativa*)	*OsDi19-2*	LOC_Os01g73960
Rice (*Oryza sativa*)	*OsDi19-3*	LOC_Os02g20170
Rice (*Oryza sativa*)	*OsDi19-4*	LOC_Os05g01730
Rice (*Oryza sativa*)	*OsDi19-5*	LOC_Os05g28980
Rice (*Oryza sativa*)	*OsDi19-6*	LOC_Os05g48800
Rice (*Oryza sativa*)	*OsDi19-7*	LOC_Os12g36900

TABLE S3 The Ka/Ks value of segmental duplication gene pairs of *PvDi19s*.

Seq-1	Seq-2	Ka	Ks	Ka/Ks
Phvul.001G210800	Phvul.007G199600	0.441940623	1.552131489	0.284731433
Phvul.001G210800	Phvul.009G252700	0.545807324	1.601670908	0.340773702
Phvul.007G199600	Phvul.009G252700	0.333443219	1.745631814	0.191015778
Phvul.003G205500	Phvul.009G173300	0.34864106	0.68730078	0.507261261

TABLE S4 Prediction of phosphorylation sites in PvDi19 proteins.

Name	PKA	PKC	PKG	DNAPK	RSK	CKI	CKII
PvDi19-1	1	6	1	1	1	2	6
PvDi19-2	1	4		2		2	5
PvDi19-3	4	9	1		1	2	3
PvDi19-4	5	12	2	1		4	1
PvDi19-5	3	14			1		4
PvDi19-6	3	6	1	1	1	4	5

Name	CDC2 kinase	CDK5 kinase	MAPK	ATM	SRC	GSK3	INSR
PvDi19-1	7						1
PvDi19-2	2						
PvDi19-3	2	1	2				1
PvDi19-4	9			1			
PvDi19-5		1	1		2	1	1
PvDi19-6	12	2	1				2
