# Supplementary material for: Identification and expression of the Di19 gene family in response to abiotic stress in common bean (Phaseolus vulgaris L.)
Source: Front Genet. 2024 May 30;15:1401011. doi: 10.3389/fgene.2024.1401011 (PMC11169598; doi:10.3389/fgene.2024.1401011)
Supplement: Supplementary file 2 [file Image1.pdf]

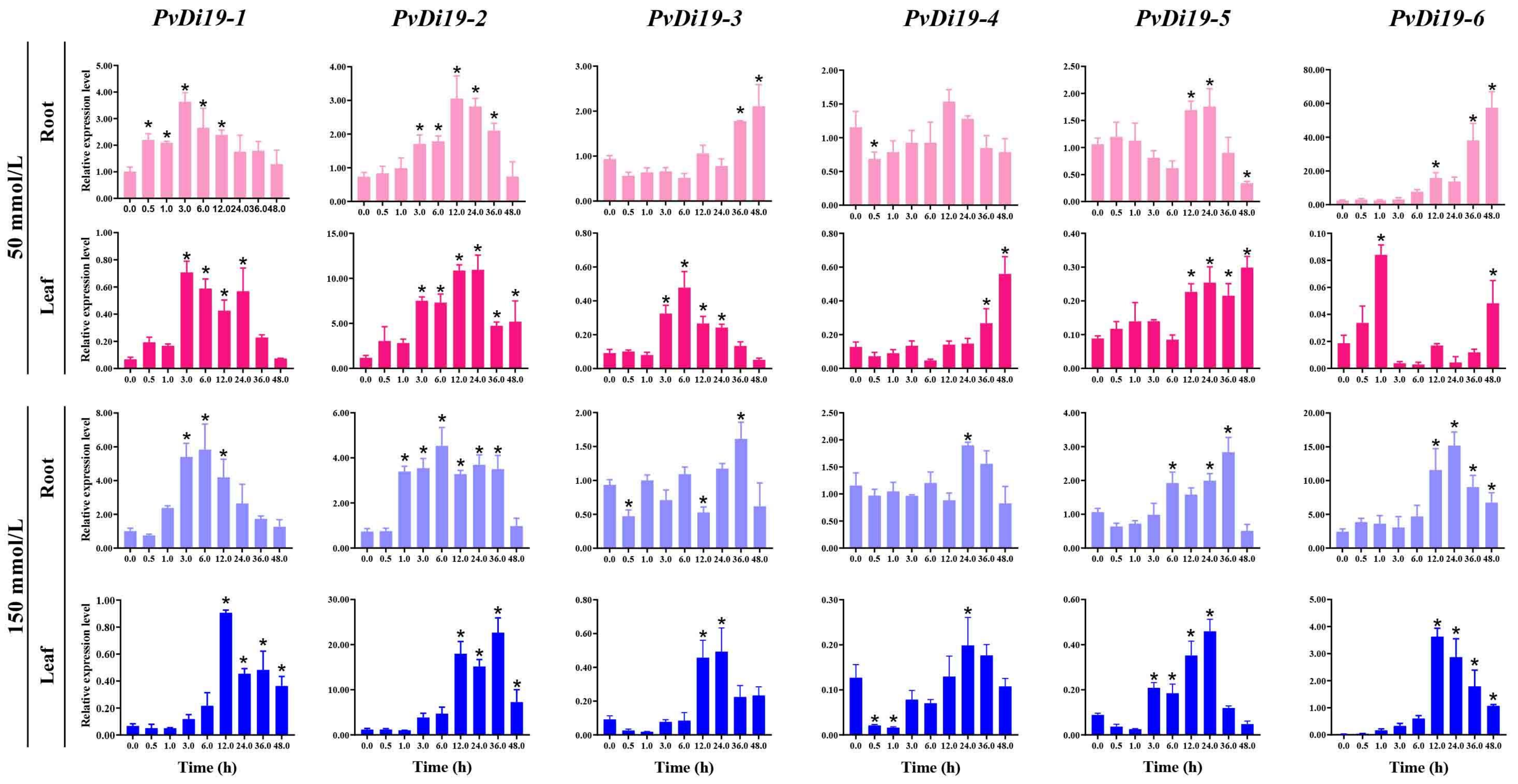


**FIGURE S1**

Expression patterns of *PvDi19s* under saline treatment. The error bars indicate standard deviation (SD) (n=3). Significance analysis is performed between the data of pre-treatment (0 h or 0 d) with each time point after treatment. *, P <0.01. The same below.

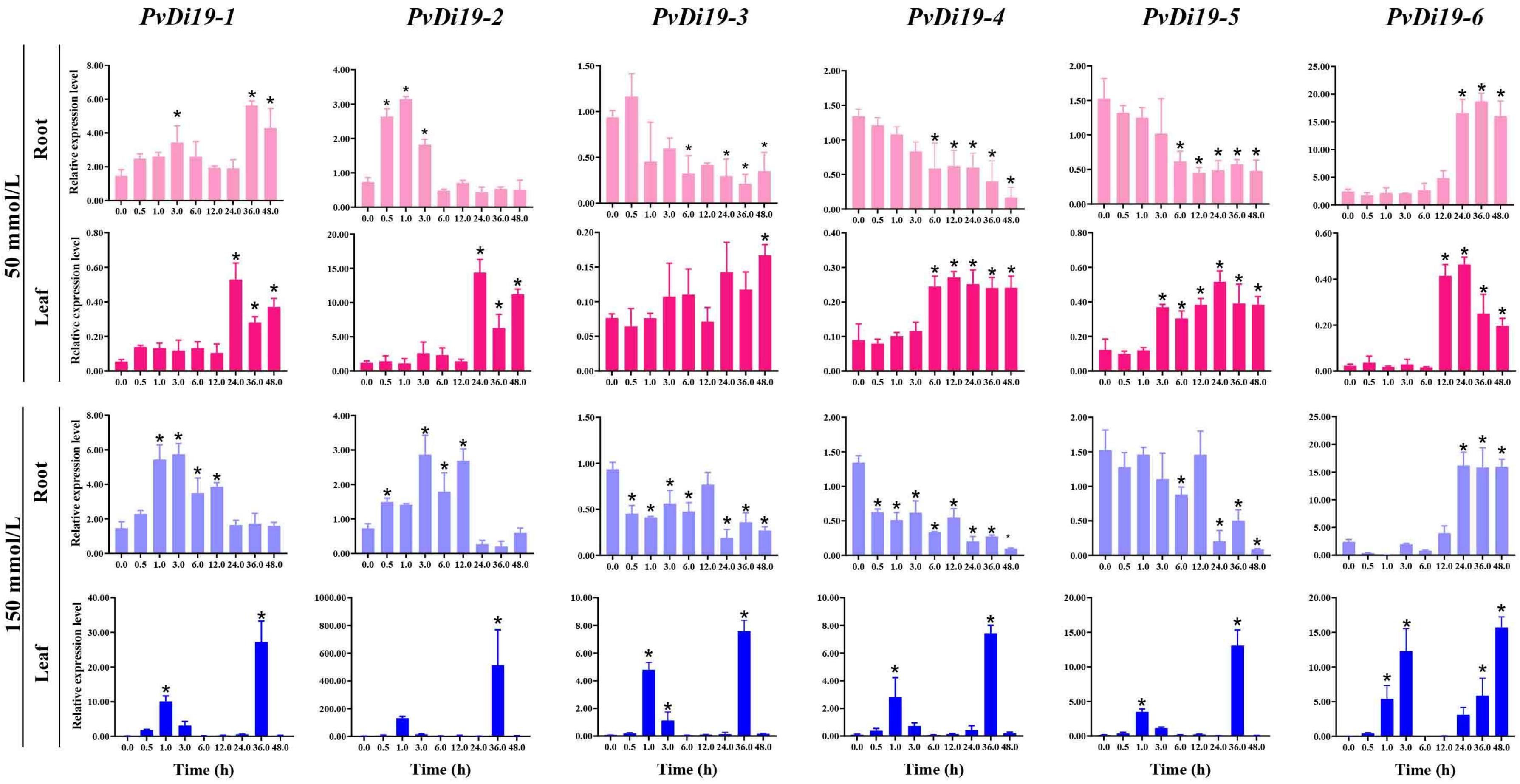


**FIGURE S2**

Expression patterns of *PvDi19s* under alkali treatment.

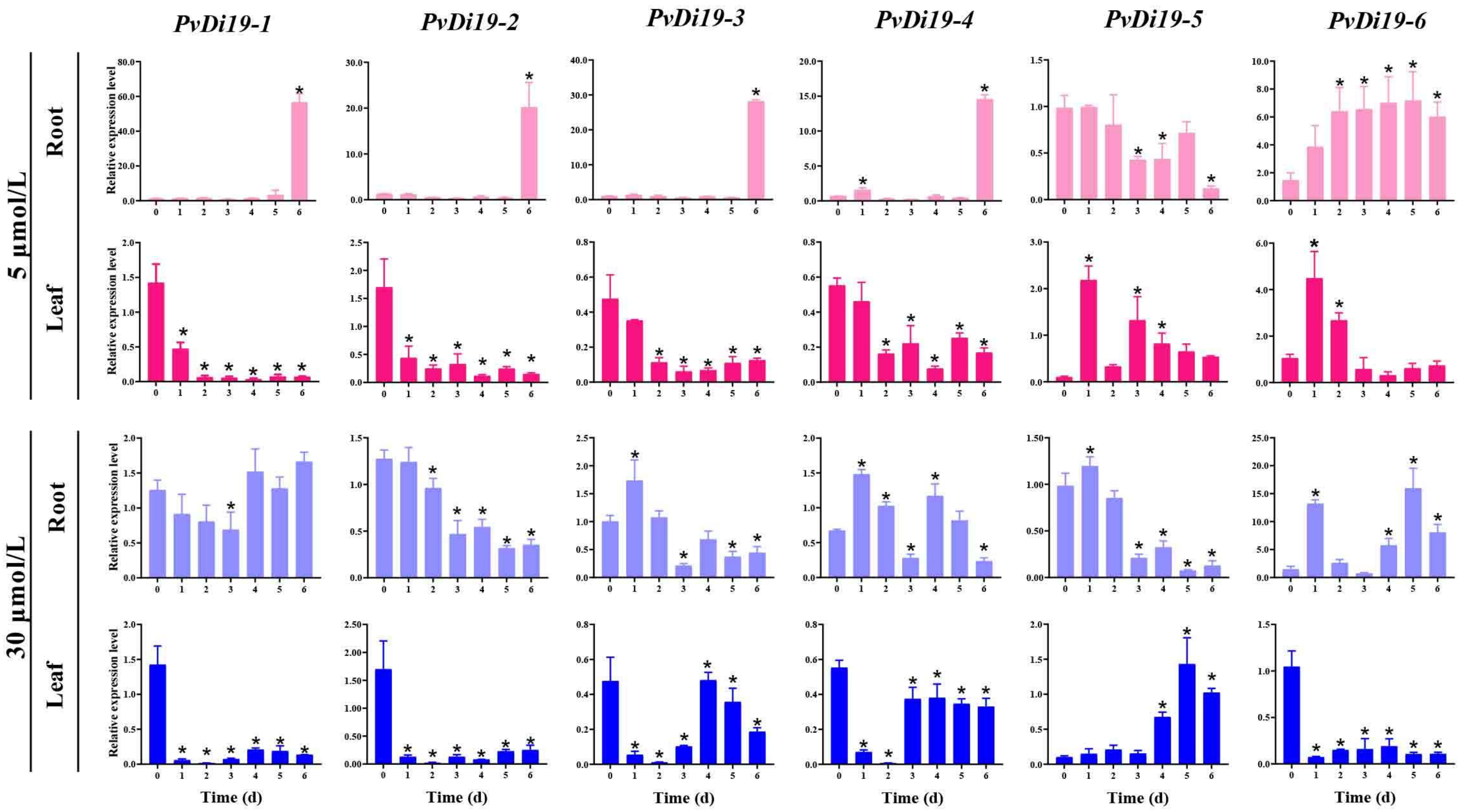


**FIGURE S3**

Expression patterns of *PvDi19s* under $CdCl_2$ treatment.

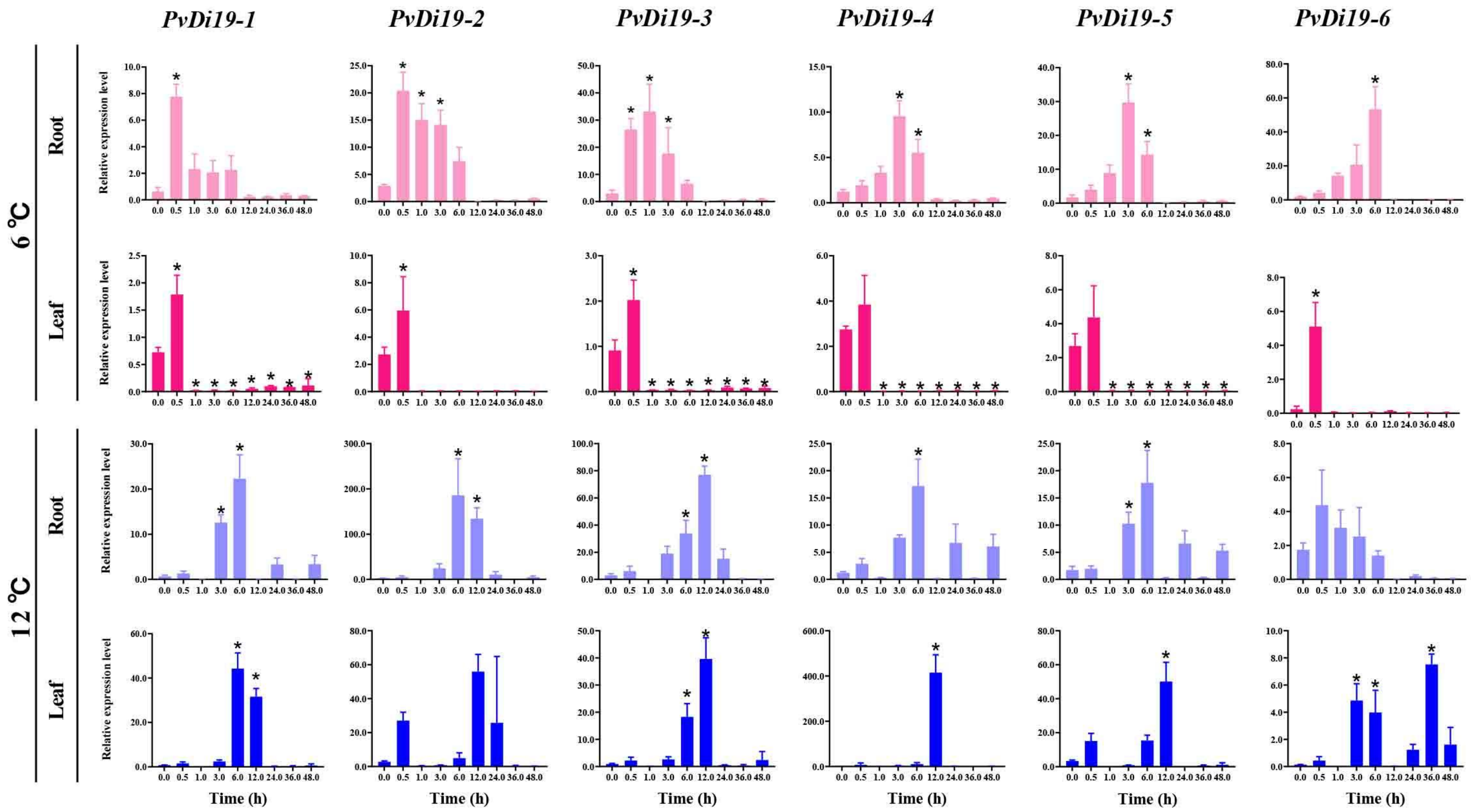


**FIGURE S4**

Expression patterns of *PvDi19s* under cold treatment.
